# STC2+ Malignant Cell State Associated with EMT, Tumor Microenvironment Remodeling, and Poor Prognosis Revealed by Single-Cell and Spatial Transcriptomics in Colorectal Cancer

**DOI:** 10.32604/or.2025.070143

**Published:** 2025-12-30

**Authors:** Kai Gui, Tianyi Yang, Chengying Xiong, Yue Wang, Zhiqiang He, Wuxian Li, Min Tang

**Affiliations:** 1 School of Laboratory Medicine, Chongqing Medical University, Key Laboratory of Clinical Laboratory Diagnostics, Ministry of Education, Chongqing, 400016, China; 2 Department of Clinical Laboratory, Women and Children’s Hospital of Chongqing Medical University, Chongqing, 401147, China; 3 Department of Clinical Laboratory, Chongqing Health Center for Women and Children, Chongqing, 401147, China

**Keywords:** Colorectal cancer (CRC), machine learning, fructose metabolism, tumor microenvironment (TME), prognosis

## Abstract

**Objectives:**

The mechanism by which specific tumor subsets in colorectal cancer (CRC) use alternative metabolic pathways, particularly those modulated by hypoxia and fructose, to alter the tumor microenvironment (TME) remains unclear. This study aimed to identify these malignant subpopulations and characterize their intercellular signaling networks and spatial organization through an integrative multi-omics approach.

**Methods:**

Leveraging bulk datasets, single-cell RNA sequencing, and integrative spatial transcriptomics, we developed a prognostic model based on hypoxia-and fructose metabolism-related genes (HFGs) to delineate tumor cell subpopulations and their intercellular signaling networks.

**Results:**

We identified a specific subset of stanniocalcin-2 positive (STC2+) malignant cells spatially enriched within tumor regions and strongly associated with poor prognosis. This subset served as a key signaling hub in the TME, exhibiting increased epithelial–mesenchymal transition activity. STC2+ cells engage in two spatially organized ligand–receptor interactions: the growth differentiation factor 15 (GDF15)—transforming growth factor beta receptor 2 (TGFBR2) pathway targeting endothelial cells and the migration inhibitory factor (MIF)—(cluster of differentiation 74 [CD74]+C-X-C motif chemokine receptor 4 [CXCR4]) pathway targeting macrophages.

**Conclusion:**

This study identified a malignant cell state in CRC that is metabolically defined and spatially limited, including liver metastases, and is characterized by elevated STC2 expression and active immune-stromal interactions. Given the interplay between metabolic reprogramming and TME remodeling, STC2+ malignant cells are a functionally significant subpopulation and a potential therapeutic target.

## 1 Introduction

Colorectal cancer (CRC) remains one of the leading causes of cancer-related mortality globally due to its high rate of metastasis and recurrence, despite significant advancements in early screening and treatment approaches [1,2]. The dynamic remodeling of the tumor microenvironment (TME) and metabolic reprogramming are crucial mechanisms that facilitate tumor invasion [3], immunological evasion [4], and treatment resistance [5]. Solid tumors exhibit hypoxic and nutrient-deficient microenvironments, compelling cancer cells to employ adaptive metabolic strategies to sustain growth [6]. Although aerobic glycolysis has been extensively investigated, emerging evidence suggests that fructose metabolism may serve as an alternative energy source [7]. During metabolic stress, fructose metabolism can improve biosynthetic activity and preserve redox balance by circumventing crucial rate-limiting steps of glycolysis [8,9]. However, the functional implications and prognostic importance of metabolic adaptations in CRC induced by fructose and hypoxia are unclear.

In this study, we employed the least absolute shrinkage and selection operator (LASSO)-Cox regression to develop a robust multigene prognostic model after analysis of a selected set of genes associated with hypoxia and fructose metabolism (HFGs). To further investigate this, we utilized a consensus machine learning framework that incorporated the Boruta, extreme gradient boosting (XGBoost), random survival forest (RSF), and Support Vector Machine-Recursive Feature Elimination (SVM-RFE) algorithms to identify the primary feature of this model. In addition, we employed single-cell and spatial transcriptomics to dissect the function of this feature in the context of colorectal cancer. These high-resolution omics technologies allowed us to understand the expression pattern in great detail, as well as its spatial localization in the tumor microenvironment. Through these analyses, our research relates hypoxia-fructose metabolism with TME remodeling by identifying a malignant cell state characterized by its interaction with the TME and elucidates how it promotes the malignant behavior of tumor cells. In addition, we used the eXtreme Sum (XSum) algorithm to discover potential small molecules that may inhibit the related oncogenic signaling of this malignant cell subpopulation.

In summary, this study aims to investigate the prognostic significance of hypoxia and fructose metabolism in colorectal cancers. With the indication of its key characteristics, we elucidated a distinct subpopulation of malignant cells and explored the mechanisms by which it drives the progression of colorectal cancer and how it interacts with immune cells within the tumor microenvironment. This work may offer important insights towards designing novel colorectal cancer therapies with improved clinical outcomes.

## 2 Materials and Methods

### 2.1 Data Acquisition

The Cancer Genome Atlas (TCGA, https://portal.gdc.cancer.gov/) supplied high-throughput sequencing data and related clinical information for several cancer types, including CRC. Several validation cohorts and related clinical data were obtained from the Gene Expression Omnibus (GEO, https://www.ncbi.nlm.nih.gov/geo/, accessed on 23 September 2025), including GSE17536, GSE29621, GSE41258, GSE87211, GSE161158, GSE103479, GSE39582, GSE18105, and GSE21510. Corresponding spatial transcriptomic datasets were obtained from the 10x Genomics platform (https://www.10xgenomics.com/) and a previous study (PMID: 34417225) [10]. The single-cell RNA sequencing datasets for human CRC were obtained from the TISCH2 database [11] (http://tisch.comp-genomics.org/). Supplementary Table S1 presents comprehensive dataset information. Moreover, we obtained immunohistochemistry and immunofluorescence slides from the Human Protein Atlas (HPA, https://www.proteinatlas.org/) database to assess the differential protein expression of genes and the subcellular localization features in the cell. The HFGs comprised the genes primarily derived from the HALLMARK_HYPOXIA and KEGG_FRUCTOSE_AND_MANNOSE_METABOLISM pathways in the Molecular Signatures Database (MSigDB, https://www.gsea-msigdb.org). This initial list was subsequently supplemented with additional relevant genes identified from the literature. After removing duplicates, the compiled HFGs list comprises 249 genes (Supplementary Table S2).

### 2.2 Spatial Transcriptomics and Single-Cell Data Processing

A deconvolution-based method was employed to extract high-resolution information from single-cell RNA sequencing (scRNA-seq) data, facilitating a comprehensive analysis of cell distribution and composition in spatial transcriptomics datasets. The SpatialFeaturePlot function from the “Seurat” package (version 5.3.0) [12] was utilized to spatially visualize gene expression levels by integrating gene expression profiles with spatial coordinates and presenting the results as dot plots. Spatial microregions were categorized using the deconvolution results into normal regions, which have a proportion of 0 malignant cells; mixed malignant regions, which have intermediate proportions; and malignant regions, which have a proportion of 1 malignant cell [13]. In addition, the ‘AUCell’ package (version 1.28.0) [14] was employed to quantify the enrichment of HFGs’ activity within each spatial microregion. The TISCH2 database supplied the human CRC scRNA-seq datasets, which subsequently underwent standard quality control procedures. First, we applied the following quality control criteria: mitochondrial gene percentage (percent.mt) ≤ 20%, nCount_RNA ≥ 1000, and nFeature_RNA between 200 and 10,000. Subsequently, the LogNormalize method was employed to standardize the data. Uniform manifold approximation and projection (UMAP) was employed to reduce dimensionality after batch effects were corrected using the “Harmony” package (version 1.2.3) [15]. The “pheatmap” package (version 1.0.13) was utilized to generate heatmaps, and the “Nebulosa” package (version 1.16.0) was employed to estimate the gene expression density across cell populations for visualization [16]. The “CellChat” software (version 1.6.1) was employed to analyze cell-cell communication to infer intercellular signaling networks [17].

### 2.3 Construction of a Prognostic Risk Scoring Model

A prognostic gene signature was created through a two-step process. First, genes possessing prognostic significance were screened using univariate Cox regression analysis. The selected candidates underwent a LASSO regression analysis to develop a final model that was robust and non-overfit. This was achieved using the “lambda.min” criterion and the “glmnet” package (version 4.1-9) [18]. The risk score for each patient with CRC was calculated using a method in which gene expression levels were weighted by their LASSO coefficients: the $\sum_{i=1}^{n}(GeneExpressioni\times Coefi)$. “*n*” represents the total number of genes included in the prognostic model. “*i*” represents the index, which iterates from 1 to *n*, allowing the equation to sum up the contributions of each individual gene in the model.

### 2.4 Analysis of Gene Expression Differences among Risk Groups

Differential expression analysis across risk groups was conducted using “Deseq2” (version 1.46.0) [19]. Genes with an absolute log2 fold-change >1 and an adjusted *p*-value (Padj) <0.05 were considered differentially expressed genes (DEGs). Results were illustrated using volcano plots generated with the “ggpubr” package (version 0.6.1).

### 2.5 Biological Function Analysis

We performed pathway enrichment analysis. In particular, we conducted Kyoto Encyclopaedia of Genes and Genomes (KEGG) pathway enrichment analysis and Gene Ontology (GO) enrichment analysis on the DEGs list through the “ClusterProfiler” package (version 4.14.6) [20]. Moreover, in order to identify the important biological processes without depending on certain thresholds, gene set enrichment analysis (GSEA) was conducted. All expressed genes were sorted by their log2 fold change values, and the sorted list was analyzed using the HALLMARK gene set. Visualizations were generated using the “ggplot2” R package (version 3.5.2).

### 2.6 Prognostic Performance Evaluation and Identification of Key Genes

Kaplan–Meier survival analysis was used to compare overall survival between patient subgroups stratified by the risk model. Time-dependent receiver operating characteristic (ROC) curves were generated using the “timeROC” (version 0.4) package [21] to assess prognostic performance. To identify the most influential gene within the prognostic signature, we implemented a consensus-based machine learning approach that integrates four independent algorithms. We employed this combined consensus to mitigate algorithm-specific bias and improve feature robustness. Features consistently ranked high across methods were considered biologically meaningful. The TCGA-CRC cohort was randomly split into training and testing sets of equal size, and a suite of four independent machine learning algorithms—XGBoost, SVM, Boruta feature selection, and RSF—was employed on the training set to evaluate feature importance. Each algorithm generated importance scores for the genes. The gene with the highest overall consensus score was designated as the key gene within the prognostic model and was selected for further functional and mechanistic investigation.

### 2.7 Cell Cultivation and Quantitative Reverse Transcription Polymerase Chain Reaction (qRT-PCR)

Human CRC cell lines SW480 (CL-0223B), Caco2 (CL-0050), HCT116 (CL-0096), LoVo (CL-0144), SW620 (CL-0225B), and normal human colon mucosal epithelial cells (NCM460) (CL-0393) were obtained from Procell Life Science & Technology Co., Ltd. (Wuhan, China). All cell lines tested negative for mycoplasma contamination using PCR-based assays, and their identities were confirmed by short tandem repeat (STR) profiling. The cells were cultivated at 37°C in a humidified incubator with high-glucose DMEM (Gibco, Cat# C11995500BT, Waltham, MA, USA), supplemented with 10% fetal bovine serum (Lonsera, Cat# S711-001S, South American Origin, Montevideo, Uruguay) and 1% penicillin-streptomycin (Biosharp, Cat# BL505A, Hefei, China). The SteadyPure Quick RNA Extraction Kit (Accurate Biotechnology, Cat# AG21023, Changsha, China) was utilized to extract total RNA from colorectal cancer cell lines, and the Evo M-MLV RT Mix Kit (Accurate Biotechnology, Cat# AG11728, Changsha, China) was employed for reverse transcription. The SYBR Green Premix PRO Taq HS qPCR Kit (Accurate Biotechnology, Cat# AG11701, Changsha, China) was employed to perform qRT-PCR. GAPDH was used as an internal control. Primers were designed and synthesized using Sangon Biotech (Shanghai, China). Supplementary Table S3 presents their sequences.

### 2.8 Cell Counting Kit-8 (CCK-8) and Colony Formation Assays

HCT116 and LoVo CRC cell lines were cultured under normoxic conditions (37°C, 5% CO_2_) in a standard incubator, and under hypoxic conditions (1% O_2_) using an AnaeroPack system (Cat# D-04, Mitsubishi Gas Chemical, Tokyo, Japan). Following the passage, cells were seeded into 96-well plates at 3000 cells/well for LoVo and 8000 cells/well for HCT116. They were subsequently incubated in normoxic and hypoxic environments. Following confirmation of adherence, culture media containing 10 µM of either sorafenib (TargetMol, Cat# 284461-73-0, Boston, MA, USA), irinotecan (TargetMol, Cat# 97682-44-5, Boston, MA, USA), or oxaliplatin (TargetMol, Cat# 61825-94-3, Boston, MA, USA) were added, and cells were incubated for an additional 24 h. Subsequently, each well received 100 µL of culture medium containing 10% CCK-8 reagent (MedChemExpress, Cat# HY-K0301, Monmouth Junction, NJ, USA), and the wells were incubated for 2 h. The Thermo Scientific Multiskan GO microplate reader (Thermo Fisher Scientific, Waltham, MA, USA) was utilized to measure absorbance at 450 nm. Additionally, 1000 HCT116 or LoVo cells were seeded into six-well plates for the colony formation test. Following colony formation and cell adherence, the medium was changed out for new media that contained the same amounts of sorafenib, irinotecan, or oxaliplatin. For 7–14 days, cells were continuously cultured. Following 1% paraformaldehyde fixation (Biosharp, Cat# BL539A, Hefei, China), colonies were stained with 0.1% crystal violet (Solarbio, Cat# C8470, Beijing, China).

### 2.9 Immunological Function Analysis

We utilized the Tumor Immune Dysfunction and Exclusion (TIDE) algorithm [22] (https://tide.dfci.harvard.edu/) to assess the predictive value of the risk model for immunotherapy outcomes after immune checkpoint inhibitor (ICI) therapy. TIDE scores, reflecting T cell dysfunction and exclusion, and microsatellite instability (MSI) were calculated for each patient to evaluate immune evasion potential. We examined whether patients in the high-risk group exhibited higher scores, indicative of immune resistance. To further elucidate the predominant immune escape patterns across risk groups, the dysfunction and exclusion components were assessed independently. Additionally, the “IOBR” package (version 0.99.0) was utilized to assess immune microenvironmental features and tumor-associated metabolic activity based on predefined gene signatures [23]. Patients were stratified into quartiles (Q1, highest expression; Q4, lowest) to evaluate the immune and genomic landscape associated with STC2 expression. Average scores of several signatures pertaining to immune response and genome state were determined for every group and displayed as a heatmap using the “pheatmap” package (version 1.0.13).

### 2.10 Drug Sensitivity Analysis

The therapeutic efficacy of oxaliplatin, irinotecan, and the anti-angiogenic agent sorafenib in CRC was evaluated utilizing drug response profiles using the Genomics of Drug Sensitivity in Cancer (GDSC2; https://www.cancerrxgene.org) database. Spearman’s correlation analysis was conducted between STC2 expression levels and the area under the dose–response curve (AUC) and half-maximal inhibitory concentration (IC_50_) values using the GDSC2 dataset, Cancer Therapeutics Response Portal (CTRP; https://portals.broadinstitute.org/ctrp.v2.1/ accessed on 01 January 2025), and the PRISM Repurposing dataset (https://depmap.org/repurposing/, accessed on 23 September 2025). A connectivity map (cMAP) analysis (https://clue.io/) was performed to identify candidate compounds that could counteract STC2-driven oncogenic effects. Using the XSum (eXtreme Sum) algorithm, a feature-matching technique intended to identify agents with inverse transcriptional effects, gene expression profiles linked to STC2 were compared to cMAP perturbation signatures [24]. This analytical strategy was founded on tried-and-true methods. Compounds with lower XSum scores were believed to be promising candidates to counteract STC2’s tumor-promoting activity.

### 2.11 Somatic Mutation and Copy Number Variation Analysis

Single-nucleotide variant (SNV) data for 33 different cancer types were obtained from the TCGA database. Coding region SNVs in STC2, MIF, CD74, CXCR4, GDF15, and TGFBR2 were analyzed to determine their somatic mutation frequencies. Mutation frequency was calculated as the percentage of tumors harboring coding-region mutations relative to the total number of samples. The oncoplot function from the “maftools” package (version 2.22.0) was utilized to visualize SNV patterns [25]. GISTIC2 scores were examined to learn more about the somatic copy number variation (CNV) landscape of these six genes. A diploid (normal) copy number was believed to be indicated by a GISTIC2 score of 0. Copy number amplification was defined as scores > 0.05, while copy number deletion was defined as scores < −0.05.

### 2.12 Pathway Activity Analysis

Protein expression data were obtained from the Cancer Proteome Atlas [26] (TCPA, https://tcpaportal.org/). Activity scores for 10 canonical cancer pathways, including TSC/mTOR, epithelial—mesenchymal transition (EMT), and cell cycle pathways, were calculated using established methods [27]. The Wilcoxon test was used to assess group differences in pathway activities to ascertain the association with STC2.

### 2.13 Statistical Analysis

Data normality was assessed before hypothesis testing. For two-group comparisons, normally distributed data were analyzed using two-tailed Student’s *t*-tests, while the Wilcoxon rank-sum test was employed for non-normally distributed data. For comparisons across three or more groups, normally distributed data were subjected to a one-way analysis of variance (ANOVA), and non-normally distributed data were subjected to the Kruskal-Wallis rank-sum test. Every *in vitro* experiment was performed independently in triplicate. Data are presented as mean ± standard deviation. R (version 4.4.2) and GraphPad Prism (version 10.2.1, GraphPad Software, LLC, San Diego, CA, USA) software were utilized to create the graphs. The following levels of significance were indicated: *p < 0.05; **p < 0.01; ***p < 0.001; ****p < 0.0001.

## 3 Results

### 3.1 Enrichment Scores, Prognostic Model Construction, and Nomogram Development Based on HFGs

**Figure 1:**
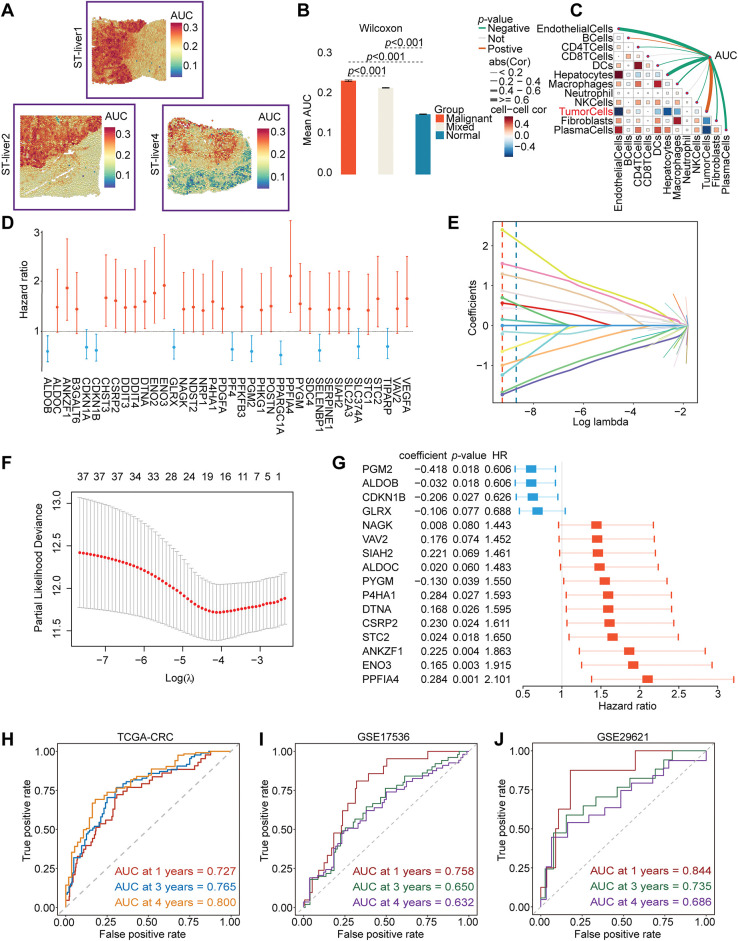
Identification and validation of a prognostic model based on HFGs in CRC. (**A**–**C**) Spatial transcriptomic analysis of CRC liver metastasis indicates that the activity of HFGs is significantly higher in malignant regions and correlates with the tumor cells. (**D**) Forest plot of the 38 genes found to be significantly associated with overall survival through the initial univariate Cox regression analysis. (**E**) The LASSO coefficient profiles of the 38 prognostic candidate genes. Each curve corresponds to one gene, and the vertical dashed line is the optimal lambda (lambda. min) selected via cross-validation. Genes with a non-zero coefficient at this value were considered in the selected model. (**F**) The 10-fold cross-validation figures for selecting the best penalty parameter (λ). (**G**) Forest plot of hazard ratios of 16 final genes of the prognostic signature. (**H**–**J**) Time-dependent ROC curves demonstrating the predictive performance of the prognostic model

**Figure 2:**
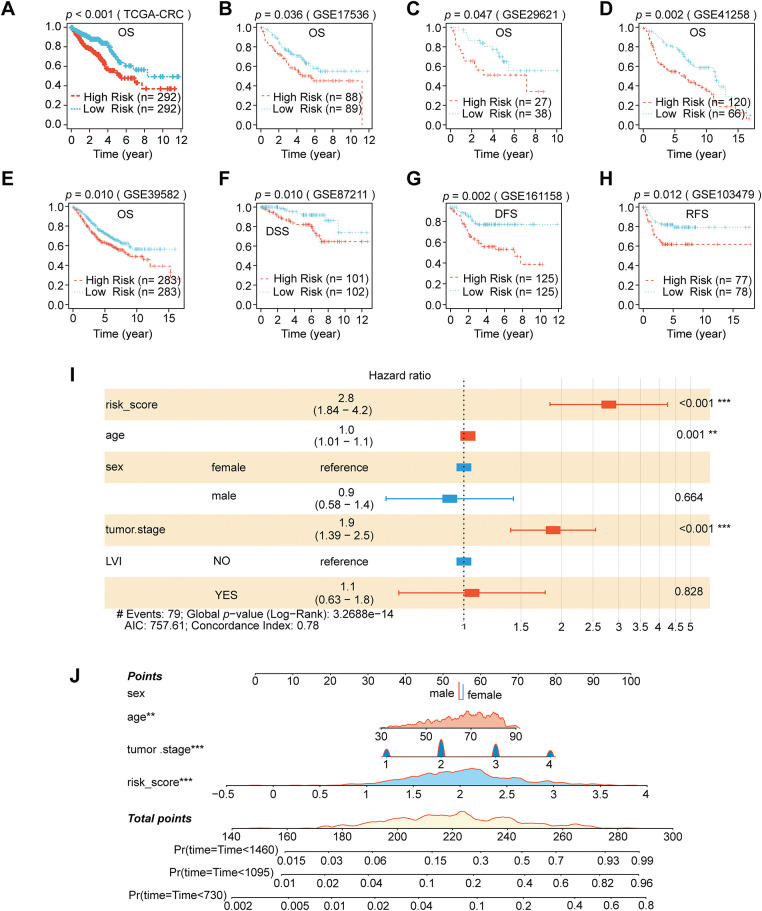
Nomogram development and prognostic performance in CRC. (**A**–**H**) Kaplan–Meier survival analyses comparing eight separate CRC cohorts’ high-and low-risk groups categorized by the risk score. (**I**) Multivariate Cox regression analysis using clinical parameters and the risk score. (**J**) Nomogram construction that incorporates clinical variables and the risk score. (**p < 0.01; ***p < 0.001)

We performed spatial transcriptome enrichment analysis on liver metastatic CRC samples. HFGs’ activity scores were significantly more enriched in malignant regions than in normal tissue areas, according to the AUCell algorithm (Fig. 1A,B and Supplementary Fig. S1A). Furthermore, Spearman correlation analysis revealed that HFGs’ activity was largely associated with tumor cells (Fig. 1C). According to these results, HFGs might be crucial for preserving malignant phenotypes, promoting metastases, and influencing overall clinical results. Consequently, HFGs expression profiles could be employed as prognostic biomarkers in CRC. The candidates were initially reduced to 38 genes associated with overall survival (OS) using univariate Cox regression analysis (Fig. 1D). The LASSO-Cox regression algorithm produced a strong 16-gene risk signature (Fig. 1E,F). As described in the Materials and Methods, this final signature, which includes ALDOB, ALDOC, ANKZF1, CDKN1B, CSRP2, DTNA, ENO3, GLRX, NAGK, P4HA1, PGM2, PPFIA4, PYGM, SIAH2, STC2, and VAV2, was used to calculate a risk score (Fig. 1G). The prognostic model performed remarkably well and consistently. The model successfully identified patients with significantly different overall survival outcomes (OS; Fig. 2A) in the TCGA training cohort (Fig. 1H) with AUCs of 0.727, 0.765, and 0.800 at 1-, 3-, and 4-year periods, respectively. The risk score was consistently associated with worse outcomes across several endpoints, including OS, disease-specific survival (DSS), disease-free survival (DFS), and relapse-free survival (RFS) (Fig. 2B–H). This predictive power was successfully generalized across seven independent validation cohorts. Notably, the AUC values for 1-, 3-, and 4-year OS were 0.758, 0.650, and 0.632 in the GSE17536 cohort, respectively (Fig. 1I); in the GSE29621 cohort, the corresponding AUCs were 0.844, 0.735, and 0.686 (Fig. 1J). The risk score was demonstrated to be a strong prognostic factor by multivariate Cox regression, which was not influenced by conventional clinical factors, including age, sex, lymphovascular invasion (LVI), or tumor stage (Fig. 2I). Using data from the TCGA-CRC cohort, we developed a nomogram combining our novel risk score with essential clinical factors (Fig. 2J). The model includes five variables: risk score, age, sex, tumor stage and LVI. This nomogram can also assess individual patients with CRC’s 2-, 3-, and 4-year OS probabilities. The nomogram exhibited good predictive ability, with the time-dependent AUCs of 2, 3, and 4 years for OS all > 0.80 (Supplementary Fig. S2A). Furthermore, calibration curves revealed a good agreement between the nomogram-predicted survival and observed actual outcome (Supplementary Fig. S2B). Such findings indicate that our nomogram is a clinically available and valuable tool for predicting short-and long-term survival, which may be used to make personalized clinical decisions.

### 3.2 Biological Features, TME, TIDE Prediction, and Drug Sensitivity Analysis in Risk Groups

**Figure 3:**
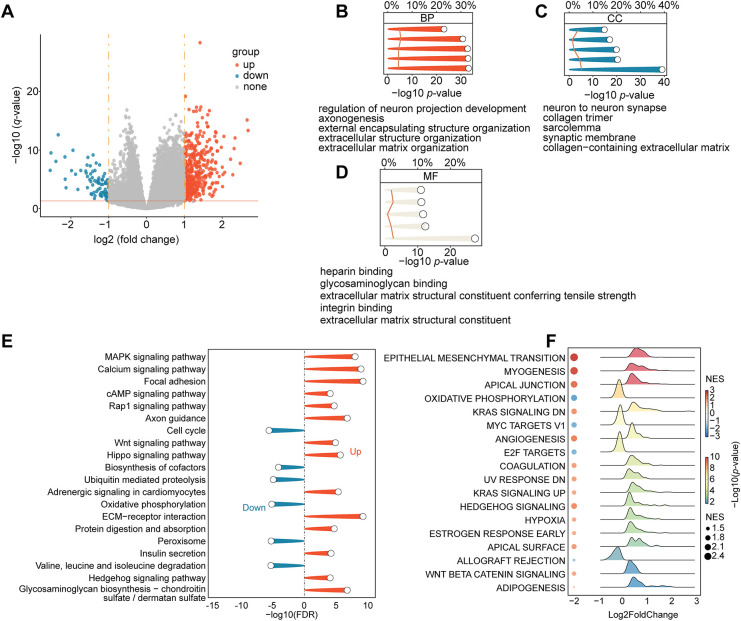

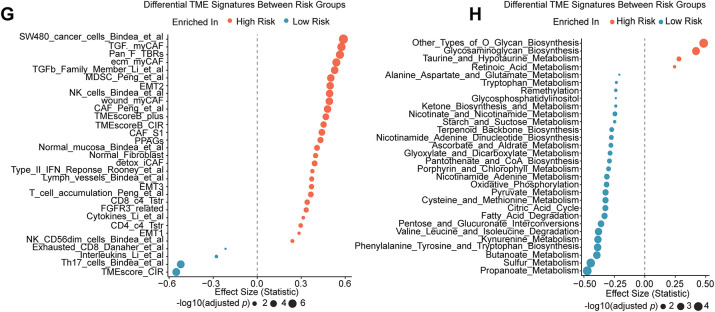
Differences in TME and functional enrichment. (**A**) Volcano plot displaying the DEGs between the high-risk and low-risk groups. (**B**–**D**) Three categories of DEGs’ GO enrichment analysis. (**E**) DEGs’ KEGG pathway enrichment analysis. (**F**) Hallmark gene sets are used in GSEA. (**G**) The “IOBR” package was used to estimate the features of the tumor immune microenvironment. (**H**) Using the “IOBR” package, different metabolic microenvironment signatures were estimated

We performed several multifaceted immunological and functional analyses. First, a study of DEGs revealed significant transcription differences between the two groups. The volcano plot revealed 497 genes that are significantly upregulated and 81 genes that are downregulated (Fig. 3A). Axon guidance, nervous system development, and extracellular matrix (ECM) organization and binding were the main processes linked to the DEGs, according to a subsequent GO enrichment analysis (Fig. 3B–D). The KEGG pathway enrichment revealed the activation of cancer-related signaling cascades, including MAPK, Wnt, and Hippo pathways, and cell adhesion (Fig. 3E). GSEA was employed to gain a more comprehensive understanding of pathway changes. High-risk tumors exhibited enhanced oncogenic pathways, including EMT, angiogenesis, hypoxia, KRAS signaling, and WNT/β-catenin signaling (Fig. 3F).

**Figure 4:**
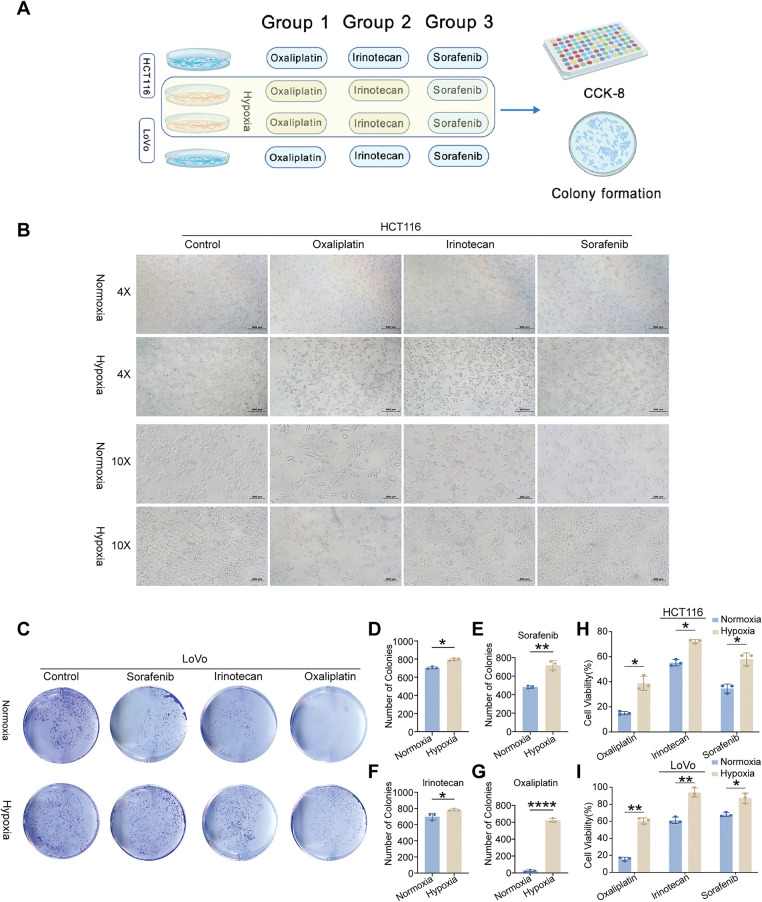
*In vitro* validation of cell viability and colony formation under normoxic and hypoxic conditions. (**A**) Schematic illustration of the experimental workflow. (**B**) Morphological and quantitative comparison of HCT116 cells treated with oxaliplatin, irinotecan, or sorafenib under normoxia and hypoxia. (**C**) Colony formation assays of LoVo cells treated with identical drug concentrations under normoxic and hypoxic conditions. (**D**) Visualization of LoVo colony counts under both oxygen conditions. (**E**–**G**) Representative image of LoVo colony formation following treatment with oxaliplatin, irinotecan, and sorafenib under normoxia and hypoxia. (**H**,**I**) CCK-8 assays revealing cell viability of HCT116 and LoVo cells treated with the three drugs under different oxygen levels. Statistical significance between normoxia and hypoxia groups was determined using a two-tailed Student’s *t*-test, *p < 0.05; **p < 0.01; ****p < 0.0001

We used the “IOBR” R package to further characterize immunological and metabolic features. Immune landscape assessment revealed that high-risk tumors have elevated levels of myeloid-derived suppressor cells (MDSCs) and TGF-β signaling components (Fig. 3G). Metabolic profiling revealed that O-Glycan Biosynthesis was upregulated in high-risk groups (Fig. 3H). The TIDE analysis validates our hypothesis, which predicted a poor ICI response for the high-risk group, with significantly higher overall TIDE, dysfunction, and exclusion scores, and MSI low (all *p* < 0.001; Supplementary Fig. S2C–F). Remarkably, a significantly greater percentage of high-risk patients (76.0%) were projected to fail to respond to immune checkpoint inhibitors (ICIs), compared to 56.5% of low-risk patients (Supplementary Fig. S2G). Additionally, we assessed the risk model’s ability to guide the selection of targeted and chemotherapeutic agents. Oxaliplatin sensitivity was significantly higher in the high-risk group (*p* = 0.033; Supplementary Fig. S2I), whereas no significant differences were observed for irinotecan or sorafenib (Supplementary Fig. S2H,J) according to IC_50_ estimates from the GDSC2 database. These findings indicate that patients in the high-risk group may be less receptive to standard therapeutic regimens. To further investigate the role of hypoxia—a key component of our risk signature—in mediating drug resistance, we conducted *in vitro* assays with two CRC cell lines (HCT116 and LoVo) cultured under normoxic and hypoxic conditions (Fig. 4A). Under hypoxia, oxaliplatin, irinotecan, or sorafenib treatment enhanced cell survival and caused morphological alterations, particularly in HCT116 cells (Fig. 4B). We investigated this observation using a colony formation assay, which confirmed our first conclusion: hypoxic LoVo cells generated significantly more colonies than their counterparts in normal oxygen (Fig. 4C–G), indicating a considerable decrease in drug sensitivity. Furthermore, we conducted CCK-8 tests to confirm these findings, which revealed that both cell lines were significantly more resistant to the medications when oxygen was in short supply (Fig. 4H,I).

### 3.3 Identification of STC2 as the Key Prognostic Gene

**Figure 5:**
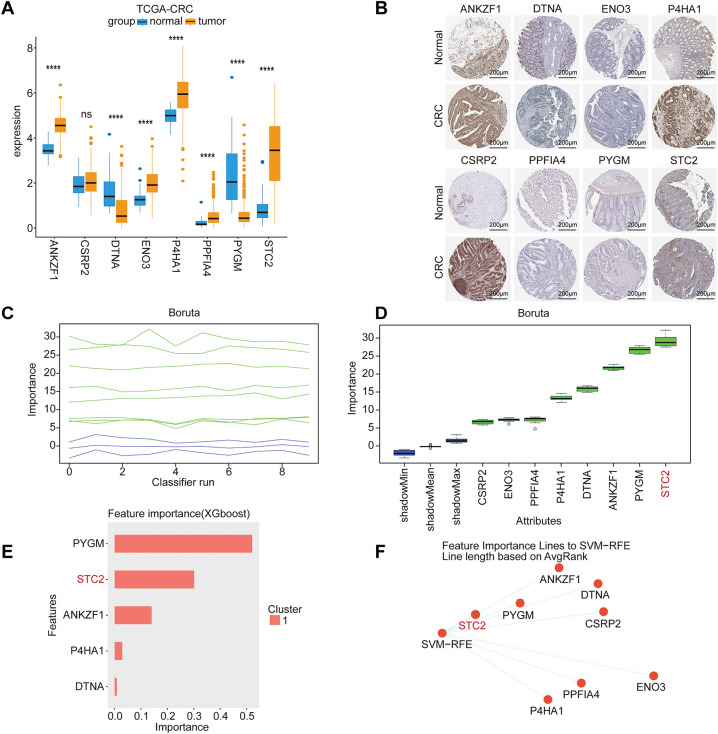

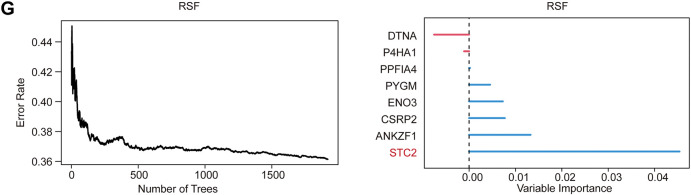
Core prognostic genes in CRC are identified using multi-algorithm machine learning. (**A**) Model gene expression differences between adjacent normal tissues and CRC tumor tissues from the TCGA-CRC cohort. (**B**) Typical immunohistochemistry (IHC) pictures from the HPA database that indicate how STC2 is expressed in normal colon tissues as opposed to CRC tissues. (**C**) Trace plot of feature importance scores over iterations of Boruta. As reference thresholds for feature selection, the green and blue lines represent the highest and lowest importance attained by shadow attributes, respectively. (**D**) The final ranking of each feature’s importance. After consistently outperforming the shadow feature thresholds, the Boruta algorithm validated the importance of the attributes enclosed in green boxes. (**E**) XGBoost (eXtreme Gradient Boosting) model-based feature importance ranking. (**F**) Support Vector Machine-Recursive Feature Elimination (SVM-RFE) is used to rank the most predictive features. (**G**) STC2 was found to be the most significant prognostic variable among all candidates after the RSF analysis verified model stability through its converged error rate. (ns, not significant; ****p < 0.0001)

To determine the most critical gene within the 16-gene prognostic signature, we first selected eight genes with a hazard ratio (HR) > 1.5 and statistically significant *p*-values. We subsequently confirmed their dysregulated expression at the transcriptomic and protein levels using data from TCGA and the HPA, respectively (Fig. 5A,B). To identify the core gene playing the most decisive role, we implemented a consensus strategy that integrated four distinct machine learning algorithms: Boruta (Fig. 5C,D), XGBoost (Fig. 5E), SVM-RFE (Fig. 5F), and RSF (Fig. 5G). The robust consensus across this diverse suite of algorithms strongly supports STC2 as the critical component of the prognostic signature; hence, it was selected for subsequent comprehensive mechanistic investigation.

### 3.4 Comprehensive Analysis of STC2 Expression, Subcellular Localization, and Genetic Alterations in CRC

**Figure 6:**
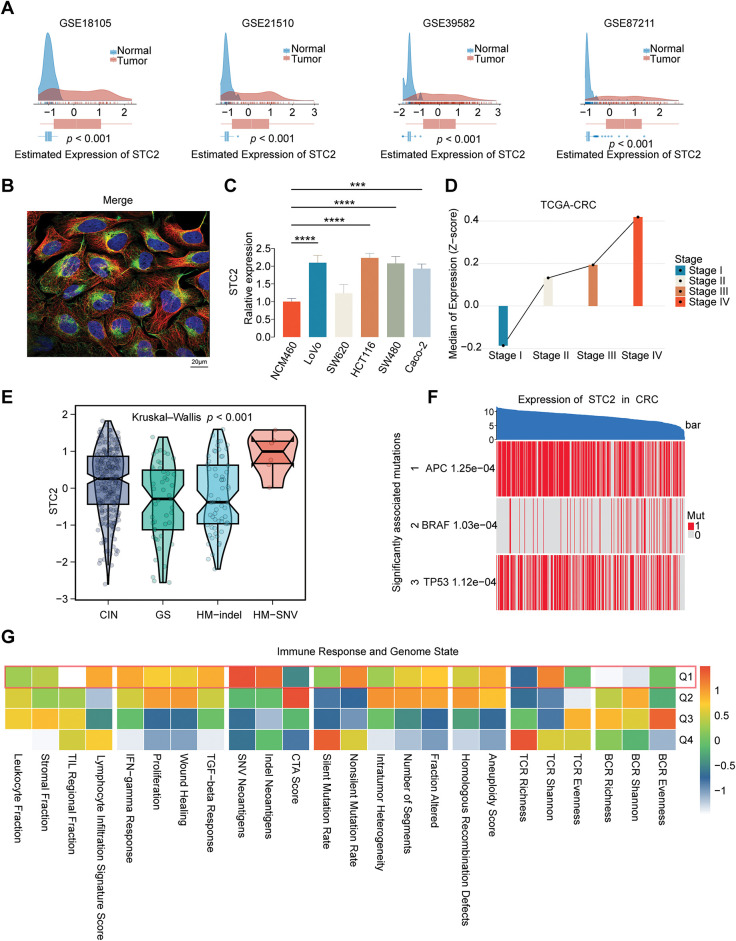
Multifaceted description of STC2 expression and how it relates to molecular and clinical characteristics in CRC. (**A**) STC2 mRNA expression density plots between tumor and normal tissues from four different GEO datasets (GSE18105, GSE21510, GSE39582, and GSE87211). (**B**) Immunofluorescence illustrating the subcellular location of STC2. Following the standard four-color code of HPA, the staining displays the target protein (STC2) in green, microtubules in red, and the nucleus in blue. (**C**) A comparison of the normal colon epithelial cell line NCM460 with a panel of CRC cell lines’ relative expression of STC2. (**D**) The TCGA-CRC cohort’s median expression of STC2 (Z-score) indicates correlation with the progression of the disease across the various clinical stages (Stages I–IV). (**E**) Violin plots illustrating STC2 expression levels among four distinct genomic instability subtypes: chromosomal instability (CIN), genomically stable (GS), hypermutated-indel (HM-indel), and hypermutated-single nucleotide variant (HM-SNV). The *p*-value indicates a significant difference across the groups. (**F**) Heatmap to illustrate the correlation between STC2 expression and mutation of pivotal CRC drivers (APC, BRAF, and TP53). Samples (columns) are ordered based on decreasing expression of STC2 (top bar), and each gene’s mutation status is marked (1: mutated; 0: wild-type). (**G**) Correlation heatmap indicating the association between STC2 expression quartiles (Q1 representing the highest 25% expression) and various signatures of the immune response and genome state. (***p < 0.001; ****p < 0.0001)

We conducted a thorough investigation to confirm STC2’s expression pattern, subcellular location, and mutational features in CRC. STC2 mRNA levels were consistently higher in CRC tumor tissues than in surrounding normal tissues (*p* < 0.001; Fig. 6A) when four public datasets (GSE18105, GSE21510, GSE39582, and GSE87211) were analyzed. Cell lines revealed this pattern; several CRC cell lines exhibited significantly greater STC2 expression than the normal colonic epithelial line NCM460 (Fig. 6C). Immunofluorescence labeling revealed that STC2 primarily resides in the cytoplasm (Fig. 6B). STC2 expression increased in the TCGA-CRC cohort from early to late clinical stages (Fig. 6D). This indicates that STC2 could be implicated in the onset of CRC. Subtype analysis revealed that the HM-SNV subtype was the most highly expressed, followed by the CIN subtype (Fig. 6E). Major oncogenic events were significantly associated with STC2 expression, which was positively correlated with the classical CIN signaling pathway activated by TP53 and APC mutations (Fig. 6F). Additionally, heatmap analysis revealed that tumors with increased STC2 expression exhibited stromal activation and genomic instability (Fig. 6G). These findings suggest that STC2 has a complex and context-specific expression pattern and is closely associated with genetic and epigenetic alterations in CRC.

### 3.5 Single-Cell Transcriptomic Landscape of STC2 Expression

**Figure 7:**
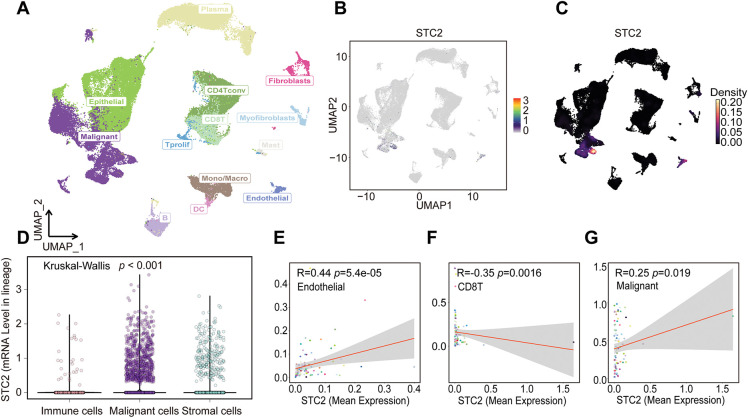

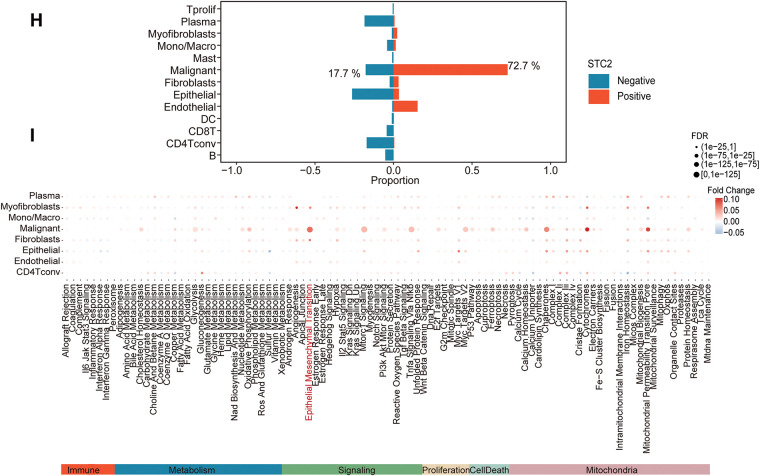
STC2 expression is intrinsic to malignant cells and is linked to TME remodeling, according to single-cell transcriptome analysis. (**A**) The single-cell transcriptome landscape from 12 CRC tumor samples is visualized using UMAP, displaying discrete cell clusters categorized by major cell types. (**B**,**C**) UMAP plots indicating STC2’s specific enrichment in malignant cells, as well as its expression level and density across all cell populations. (**D**) A violin plot comparing the expression levels of STC2 mRNA in the major cell lineages indicates that malignant cells express the gene much more than immune and stromal cells do. (**E**–**G**) Correlation analysis demonstrating the relationship between the percentage of other cell types and the mean expression of STC2 in malignant cells. (**H**) Bar graph indicating the proportionate distribution of different cell types, arranged according to the expression status of STC2. (**I**) A dot plot that illustrates how STC2-positive and STC2-negative cells differ in the activity of important biological pathways

Several cellular subpopulations were successfully identified after CRC samples’ cell types were annotated (Fig. 7A). Malignant tumor cells exhibited the highest levels of STC2 expression compared to other cell populations (Fig. 7B,C). However, immunological and stromal cell types were rarely expressed (Fig. 7D). Remarkably, correlation analyses revealed that higher STC2 expression levels in malignant cells were negatively correlated with CD8+ T cells (Fig. 7F) but positively correlated with higher proportions of endothelial and malignant tumor cells (Fig. 7E,G). Importantly, 72.7% of the STC2-positive cells were malignant compared to only 17.7% of the STC2-negative cells (Fig. 7H). This suggests that the primary source of STC2 expression may be malignant cells. Accordingly, STC2+ malignant cells were identified as this subpopulation. Cells were separated into STC2+ and STC2-groups to properly define these population pathway activity scores. According to the findings, there was a significant enrichment in EMT for the STC2+ malignant subpopulation (Fig. 7I).

### 3.6 Intercellular Interactions between STC2+ Malignant Cells, Macrophages, and Endothelial Cells

**Figure 8:**
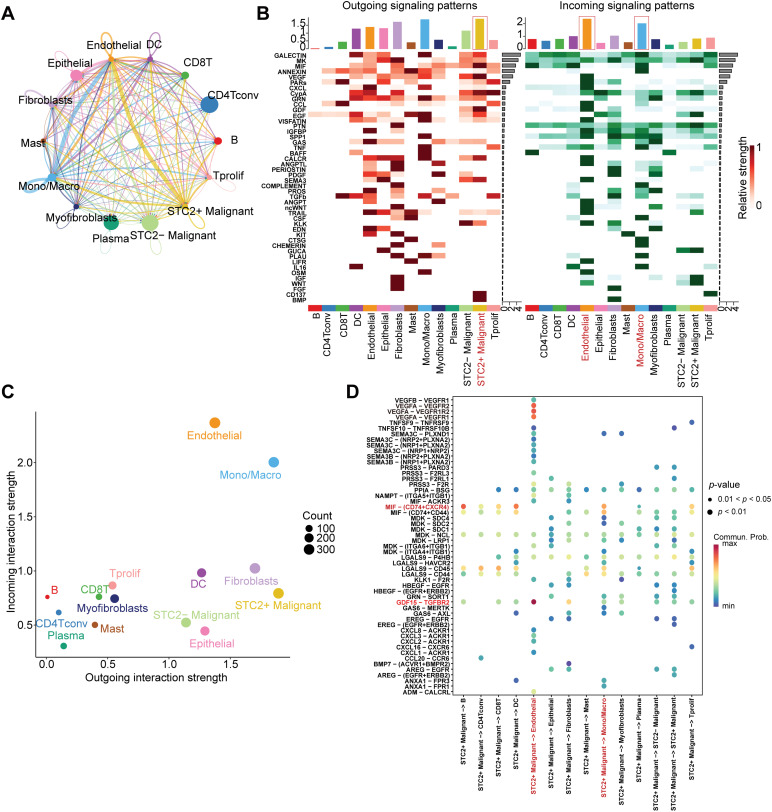

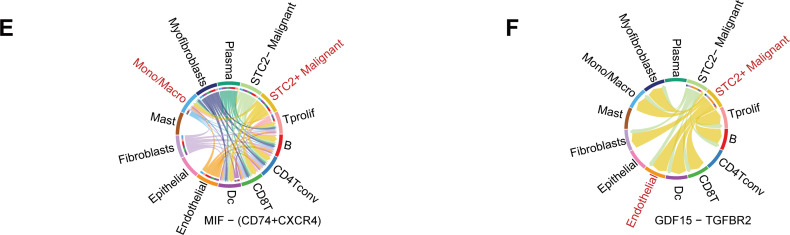
Systematic examination of the CRC microenvironment’s intercellular communication network. (**A**) A network plot that indicates the overall intercellular communication environment across all cell subtypes that have been identified. (**B**) Heatmaps indicating major pathways for various cell types’ incoming (right) and outgoing (left) signaling patterns. The primary sender and recipient cell populations are identified by the color intensity, which indicates the relative strength of the signaling. (**C**) This signal scatter plot identifies the unique communication functions of each cell subtype, emphasizing endothelial cells and macrophages as the main communication hubs within the TME and putting STC2+ malignant cells as the main signal emitters. (**D**) A dot plot that illustrates the main ligand-receptor interactions that mediate intercellular communication. The communication probability is represented by the color scale, while the dot size reflects the *p*-value significance (larger dots indicate higher significance). (**E**,**F**) Chord diagrams indicating the particular patterns of interaction for the GDF15-TGFBR2 axis and MIF-(CD74+CXCR4) axis, two chosen signaling pathways

**Figure 9:**
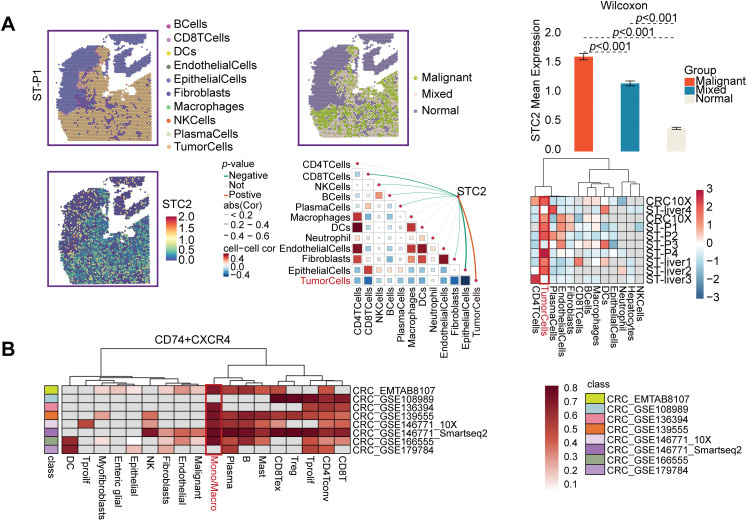

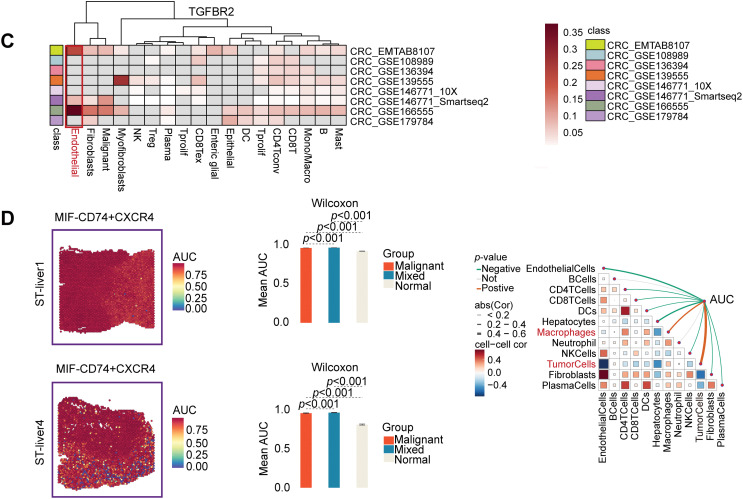
Specific communication axes driven by STC2+ malignant cells are revealed by spatial and single-cell resolution analyses. (**A**) STC2’s colocalization and enrichment in malignant regions are highlighted by the spatial maps, which also indicate the annotation of major cell types and the corresponding expression of STC2. STC2 expression is further quantified across various tissue regions in the accompanying heatmap and bar plot, which confirms that it is significantly higher within the malignant cell population. (**B**,**C**) Heatmaps indicating the expression of important receptors in several CRC single-cell RNA sequencing datasets. (**D**) The MIF–(CD74+CXCR4) axis was activated in both malignant tumor cells and macrophages, according to spatial transcriptome analysis of CRC liver metastases

**Figure 10:**
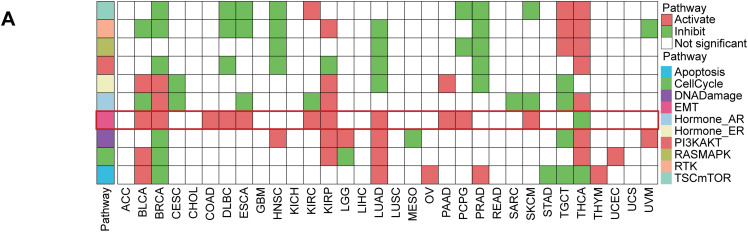

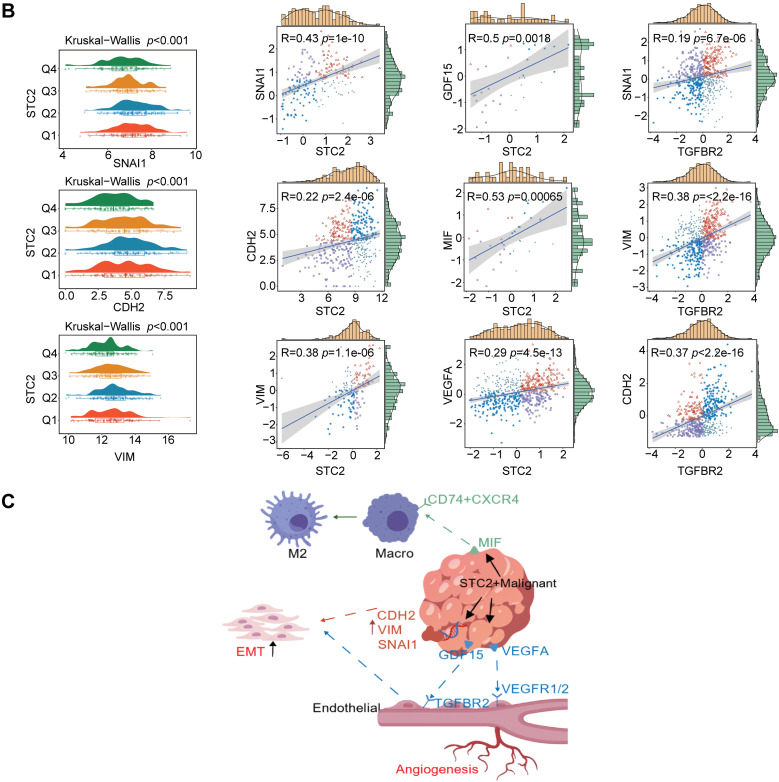
The function of STC2 is in fostering crosstalk between TME and EMT. (**A**) Pan-cancer analysis that illustrates the relationship between STC2 expression and the activity of key oncogenic and metabolic pathways in different cancer types. STC2’s strong correlation with important EMT pathways is indicated by the highlighted row for COAD (colon adenocarcinoma). (**B**) STC2 expression and important EMT markers were strongly correlated by correlation analysis. The mesenchymal markers CDH2 and VIM, as well as the transcription factor SNAI1, which drives EMT, exhibited a significant positive correlation with STC2 expression. Furthermore, MIF, GDF15, and VEGFA were positively correlated with STC2 expression. Important EMT markers were also found to correlate with TGFBR2. (**C**) Collectively, these results support the idea that STC2 participates in important signaling axes for TME remodeling and EMT regulation, such as MIF-(CD74+CXCR4), GDF15-TGFBR2, and VEGFA-VEGFR1/2

According to a detailed investigation of cell-cell interactions, STC2+ malignant cells serve as a major communication hub within the TME (Fig. 8A). These cells primarily transmit signals, whereas endothelial cells and macrophages primarily receive the signals (Fig. 8B,C). Ligand-receptor analysis revealed that STC2+ malignant cells activated the GDF15-TGFBR2 and MIF–CD74+CXCR4 signaling axes more than STC2^−^ cells (Fig. 8D–F). MIF and GDF15 ligands were consistently markedly upregulated in STC2+ malignant cells at the single-cell level (Supplementary Fig. S3A,B). According to spatial transcriptomic analysis of CRC tissues, STC2 expression was primarily found in malignant tumor regions and was substantially positively correlated with malignant cells in several CRC samples, including liver metastases (Fig. 9A, Supplementary Fig. S1B). These findings suggest that the STC2+ malignant cell subpopulation could play an important role in tumor growth. Furthermore, an integrated analysis of eight single-cell RNA sequencing datasets revealed that TGFBR2 was highly expressed in endothelial cells, whereas CD74+CXCR4 expression was strongly associated with macrophages (Fig. 9B,C). Furthermore, spatial correlation analysis revealed that the MIF-CD74+CXCR4 signaling axis was implicated in the activation of malignant cells and macrophages and was markedly elevated in the malignant regions of colorectal liver metastases (Fig. 9D). To support their possible roles in crucial oncogenic mechanisms, we assessed the mutation frequency, copy number amplification, and copy number–expression correlation of STC2, MIF, CD74, CXCR4, GDF15, and TGFBR2 across several cancer types (Supplementary Fig. S4A–C). The MIF-(CD74+CXCR4) axis is a major factor in promoting macrophage M2 polarization, according to an increasing amount of evidence from earlier research [28–30]. Therefore, we believe that malignant cells expressing STC2+ may activate this pathway to create an environment favorable to tumor growth and increase liver metastases in CRC. Previous studies have demonstrated that GDF15, as a member of the TGF-β superfamily, can activate the canonical TGF-β signaling pathway [31], stimulating and promoting the endothelium to mesenchymal transition (EndMT) [32]. This relationship is validated by our own TGFBR2 correlation analysis with EMT markers. In addition, our pathway and correlation analyses demonstrate an intensive interaction of STC2 with EMT. This relationship is validated by the fact that STC2 overexpression indicates a positive correlation with key markers of the EMT process, such as SNAI1, CDH2, and VIM. We thus speculate that the GDF15-TGFBR2 axis could act to enhance the EMT program, which seems to be endogenously executed by these STC2+ cells (Fig. 10A–C).

### 3.7 Drug Sensitivity Analysis and Targeted Therapeutic Prediction

**Figure 11:**
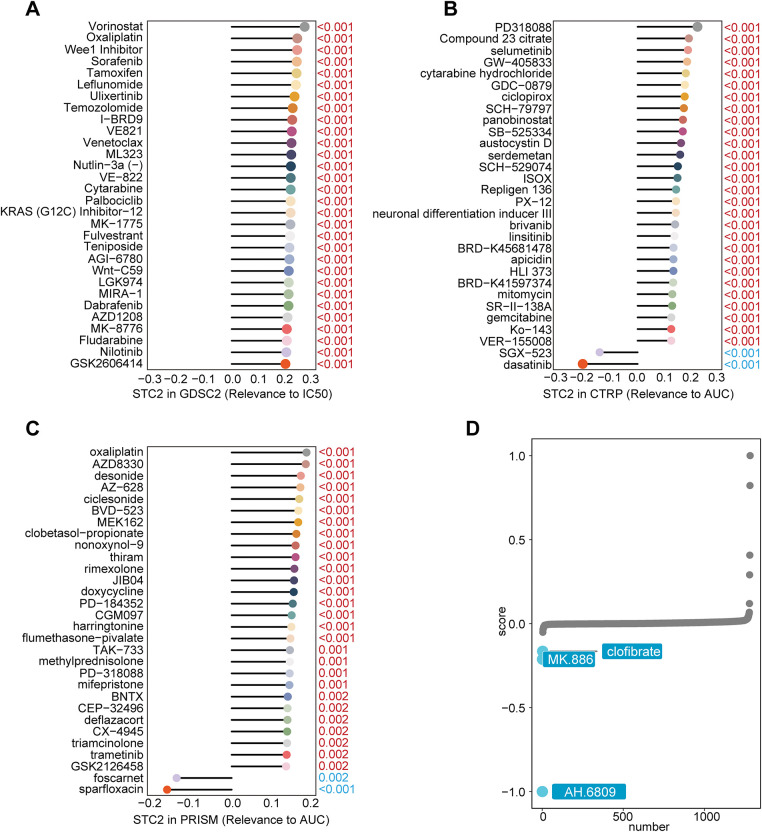
Finding possible therapeutic agents and forecasting medication response for the STC2-high malignant subgroup. (**A**–**C**) Three main pharmacogenomic databases—GDSC2, CTRP, and PRISM—are used to predict drug response in STC2-high cancer cells. The Spearman correlation between STC2 expression and drug sensitivity, as determined by the area under the AUC or IC_50_, was computed. Whereas a positive correlation implies greater resistance, a negative correlation suggests that higher STC2 expression is linked to increased sensitivity. The findings highlight the therapeutic difficulties related to this subpopulation by exposing a complex therapeutic profile for STC2-high cells, which is marked by both unique sensitivities and resistances. (**D**) Using cMAP analysis, AH-6809 was identified as a possible therapeutic agent for the STC2+ malignant subpopulation. AH-6809 emerged as a leading candidate among the small molecules identified by the analysis that were predicted to reverse the STC2-high gene expression signature

STC2 was implicated in chemoresistance after Spearman correlation analysis revealed that higher STC2 levels were generally associated with decreased drug sensitivity (higher IC_50_ or AUC values) (Fig. 11A–C). Inspired by this result, we used the XSum algorithm to perform cMAP analysis to identify compounds that could reverse the transcriptional signature of STC2-overexpression. According to this computational screen, AH-6809, a selective antagonist of the prostaglandin E_2_ receptor EP_2_, is a strong candidate to counteract the pro-tumorigenic traits associated with STC2 [33] (Fig. 11D).

## 4 Discussion

Here, we identify a functionally distinct malignant cell state in CRC characterized by elevated STC2 expression and inherently associated with a metabolic program driven by hypoxia. We propose that STC2+ malignant cell serves as a key contributor to TME remodeling as a marker of a poor prognosis. These cells function as a crucial signaling center that actively shapes a pro-tumorigenic niche through specific stromal and immune interactions, thereby establishing a connection between malignant progression and metabolic adaptation.

The TME confers a survival and invasive advantage **to cancer cells** through metabolic reprogramming [34–36]. Notably, as global fructose consumption increases, accumulating evidence indicates a strong correlation between excessive fructose consumption and the onset of CRC [37]. A defining feature of the TME is hypoxia, which facilitates metabolic reprogramming and enhances the utilization of fructose to support tumor invasion and growth [38]. Hypoxia-inducible factor-1 (HIF-1) directly upregulates the fructose-specific transporter GLUT5 to increase fructose uptake as an alternative metabolic substrate [39]. Fructose serves as an adaptive energy source under hypoxic conditions [40]. This mechanism has demonstrated efficacy across multiple cancer types. In cholangiocarcinoma, GLUT5 inhibition reduces fructose uptake and tumor cell invasion, whereas in lung cancer, it is markedly upregulated, accelerating *in vivo* tumor growth [41,42]. Furthermore, the liver is enriched with key enzymes for fructose metabolism, including Aldolase B (ALDOB). Liver metastases in CRC have been demonstrated to increase ALDOB, hence enhancing fructose utilization. A previous study has demonstrated that CRC liver metastasis can be inhibited by dietary fructose restriction or by inhibiting ALDOB expression [43].

Motivated by these results, we examined the prognostic relevance of genes associated with fructose metabolism and hypoxia in CRC. Among the spatial transcriptomic sections of CRC liver metastases, we identified that the collected HFGs were activated in malignant regions. We initiated the development of a prognostic model utilizing these HFGs, which demonstrated efficacy in predicting clinical outcomes and exhibited strong predictive performance across several independent CRC cohorts. Subsequently, we focused on STC2, the most crucial core variable in the model. We confirmed the tumor-specific expression of STC2 using spatial transcriptomics and single-cell resolution validation. Additionally, we identified a malignant subpopulation that is spatially localized and has distinct functional traits that are evidenced by STC2 expression. Spatial transcriptomic analysis revealed that this subpopulation also exhibited spatially restricted enrichment within malignant regions of CRC liver metastases. Analysis of cell-cell communication revealed that the STC2+ malignant subpopulation is an important signaling node in the TME. We discovered that this subpopulation uses the MIF–(CD74+CXCR4) and GDF15–TGFBR2 signaling pathways to facilitate interactions with other TME cell types. Previous studies indicate that MIF binds to CD74 and its co-receptor CXCR4, leading to immune cell recruitment [44], macrophage M2 polarization [29], and immunological suppression [45]. This pathway is a critical tumor–macrophage communication mechanism across various solid tumors [46–48] and represents a promising therapeutic target. Similarly, we observed markedly increased activity of the MIF–(CD74+CXCR4) axis in malignant regions of CRC liver metastases, exhibiting a strong spatial association with both macrophages and malignant cells, hence enforcing its potential influence on the metastatic microenvironment. Notably, MIF modulators commenced clinical trials [49]. The GDF15–TGFBR2 axis involves members of the TGF-β superfamily. Previous studies have demonstrated that GDF15 promotes signaling through TGFBR2 and facilitates the induction of EndMT [32]. Beyond its role in EndMT, GDF15 promotes AKT phosphorylation in a TGFBR2-dependent manner, hence increasing the resistance of esophageal squamous cell carcinoma cells to low-dose cisplatin [50]. Our pathway and correlation analyses indicate that the STC2+ cell subpopulation may exhibit aberrant EMT activation, potentially exacerbating the GDF15–TGFBR2 axis.

This study has some limitations. First, although previous studies have emphasized the importance of these two signaling pathways, we did not conduct *in vivo* or *in vitro* functional validation. Second, cMAP analysis revealed that AH-6809 (an EP_2_R antagonist) could be used as a therapeutic drug. It has been used in many preclinical studies for investigating the roles of PGE_2_ in the control of inflammation and tumor growth, but the lack of specificity of AH-6809 and its poor translational value remain as obstacles to overcome. Also, the MIF–(CD74+CXCR4) and GDF15–TGFBR2 would be challenging targets because of their broad physiological relevance and possible off-target effects. These will constitute the focus of our future research.

## 5 Conclusions

In conclusion, we propose that the STC2+ malignant cell subpopulation in CRC is metabolically reprogrammed, spatially differentiated, and immunologically interacting. We found that it may regulate the TME at the single-cell and spatial transcriptomic levels via the MIF–(CD74+CXCR4) and GDF15-TGFBR2 signaling pathways. These results offer a theoretical basis for understanding CRC progression and investigating novel treatment strategies.

## Supplementary Materials

Figure S1 Spatial transcriptomics reveals cell composition and TME heterogeneity in primary CRC and liver metastasis. (A) Spatial mapping of cell types and malignant regions in CRC liver metastasis (ST-liver1, ST-liver2, ST-liver3, ST-liver4). (B) Spatial analysis of cell types and malignancy states in primary CRC (CRC10X, ST-P2, ST-P3, ST-P4).

Figure S2 Prognostic model assessment of survival prediction, TIDE, and drug sensitivity in CRC patients. (A) ROC curves of the constructed nomogram and other clinical features for predicting 2-year, 3-year, and 4-year OS. (B) Calibration curves of the constructed nomogram. (C–F) Differences in TIDE Characteristics between high- and low-risk groups. (G) Response rates to ICI treatment in high- and low-risk groups. (H–J) Comparison of IC50 values for common chemotherapy drugs between high- and low-risk groups. (ns, not significant; ***p < 0.001)

Figure S3 Expression characteristics of MIF and GDF15 ligands in CRC single cells. (A) Single-cell expression analysis of macrophage migration inhibitory factor (MIF). (B) Single-cell expression analysis of growth differentiation factor 15 (GDF15).

Figure S4 Pan-cancer analysis reveals the landscape of genetic alterations and expression correlations of STC2, CXCR4, CD74, MIF, TGFBR2, and GDF15. (A) Gene copy number amplification ratio of the selected genes across different cancer types. (B) Oncoprint of somatic mutation distribution and SNV type classification for the selected genes. (C) Copy number and expression correlation of the selected genes.

## Data Availability

The datasets utilized in this study are publicly accessible via the repositories cited in the manuscript. Additional information is available upon request from the corresponding authors.

## Supplementary Materials

The supplementary material is available online at https://www.techscience.com/doi/10.32604/or.2025.070143/s1.
