# Supplementary figures and images for: STC2+ Malignant Cell State Associated with EMT, Tumor Microenvironment Remodeling, and Poor Prognosis Revealed by Single-Cell and Spatial Transcriptomics in Colorectal Cancer
Source: Oncol Res. 2025 Dec 30;34(1):24. doi: 10.32604/or.2025.070143 (PMC12774564; doi:10.32604/or.2025.070143)

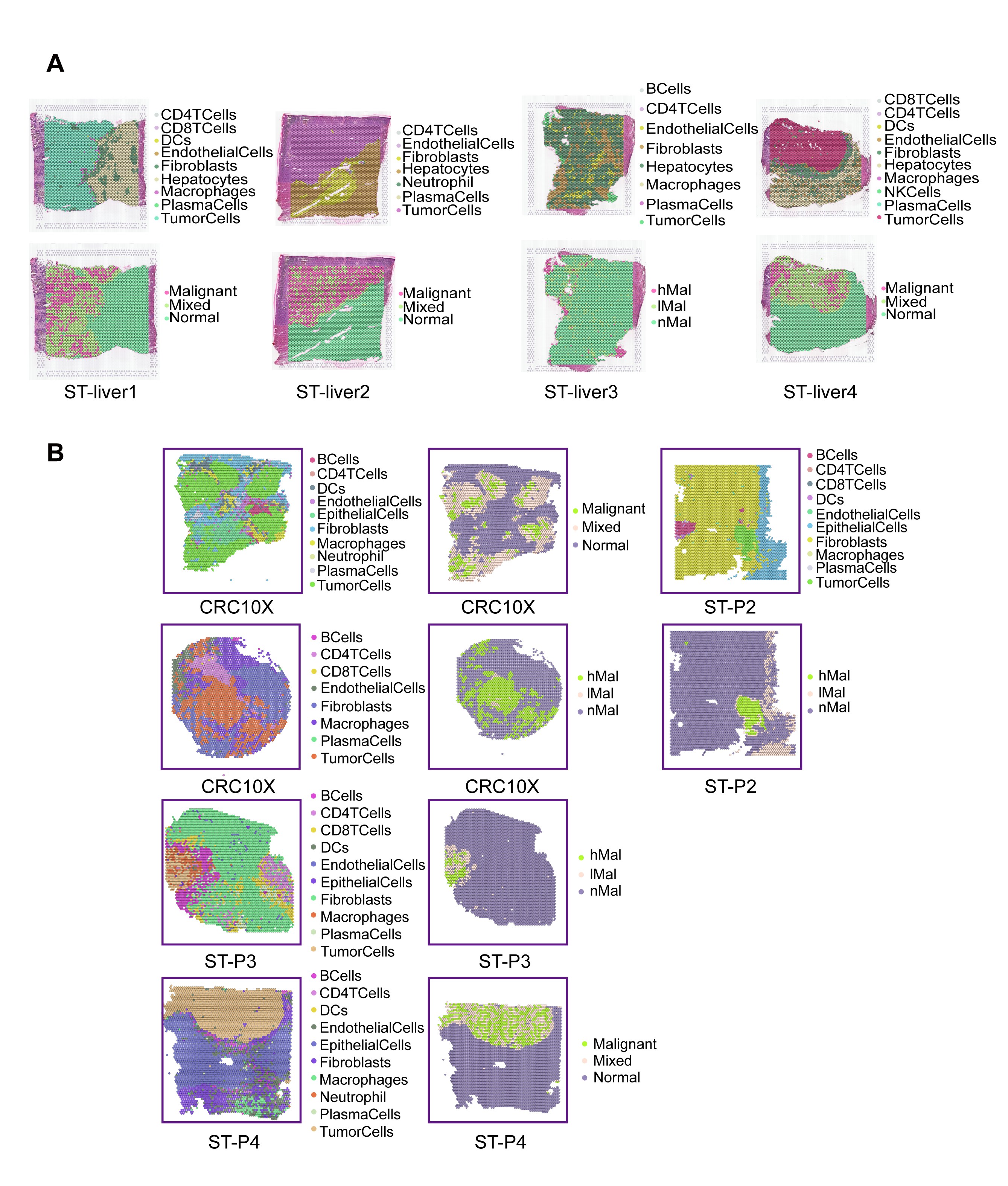

Supplement: Figure S1 [file OncolRes-34-70143-s001.tif]

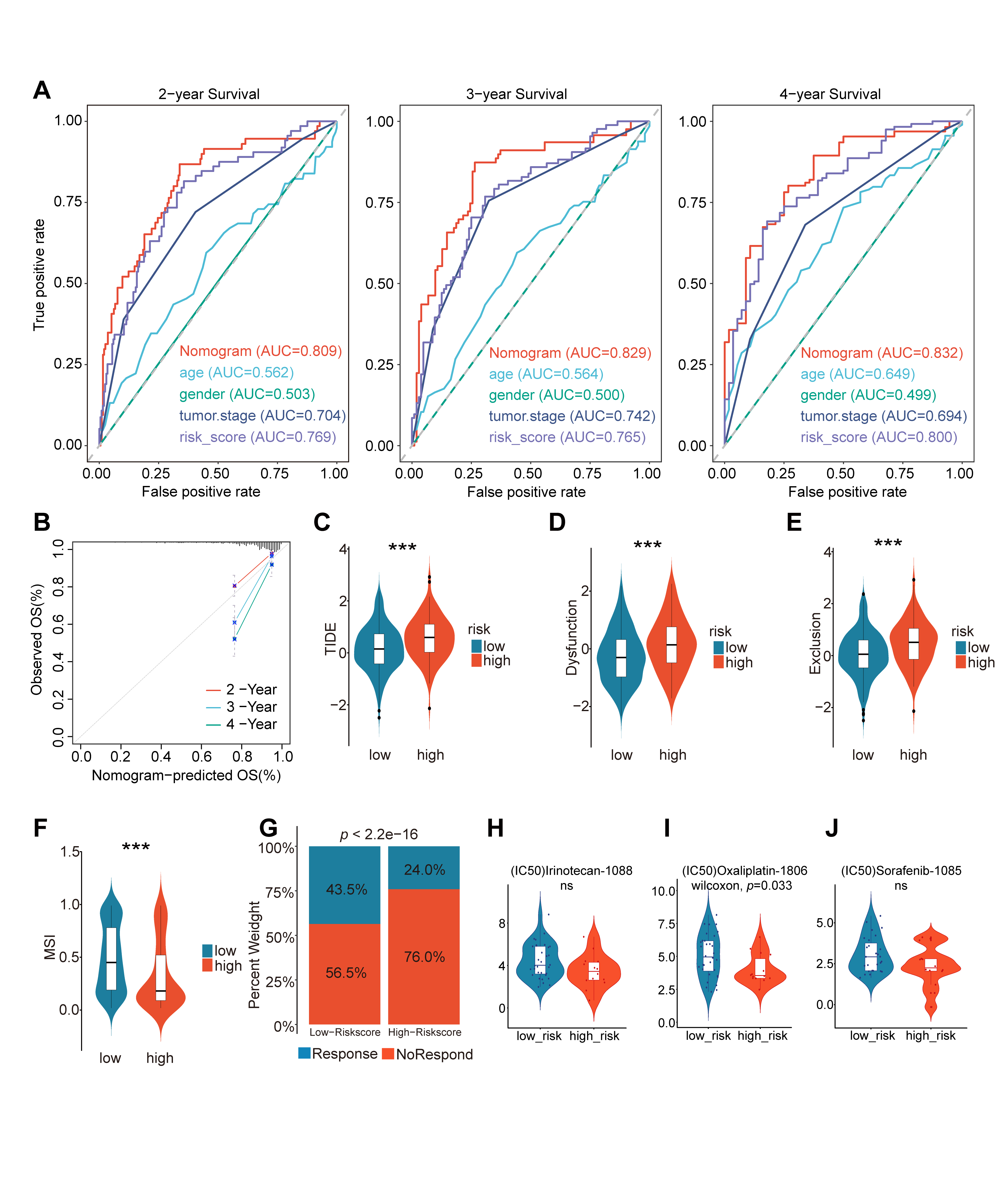

Supplement: Figure S2 [file OncolRes-34-70143-s002.tif]

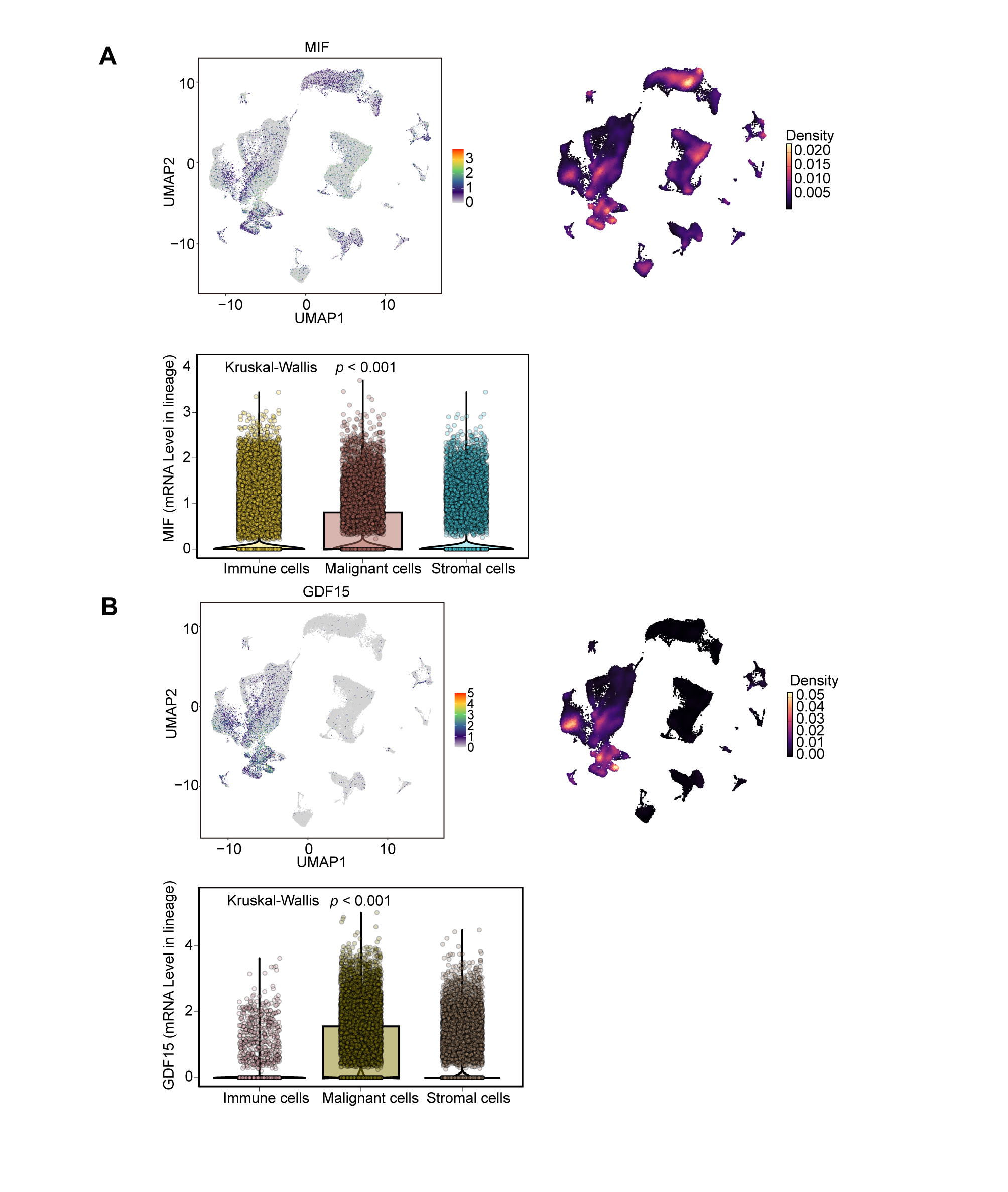

Supplement: Figure S3 [file OncolRes-34-70143-s003.tif]

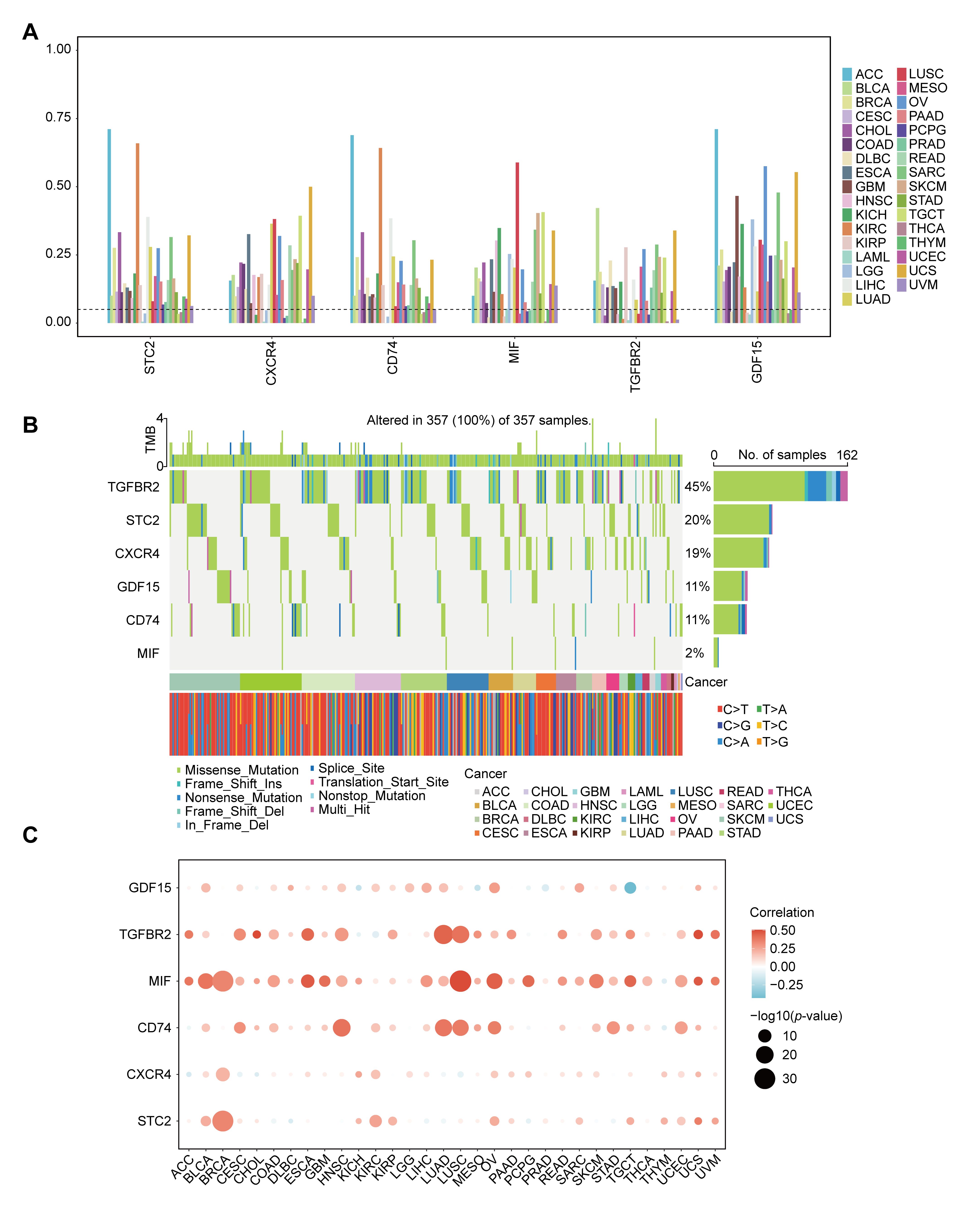

Supplement: Figure S4 [file OncolRes-34-70143-s004.tif]
