# Supplementary material for: STC2+ Malignant Cell State Associated with EMT, Tumor Microenvironment Remodeling, and Poor Prognosis Revealed by Single-Cell and Spatial Transcriptomics in Colorectal Cancer
Source: Oncol Res. 2025 Dec 30;34(1):24. doi: 10.32604/or.2025.070143 (PMC12774564; doi:10.32604/or.2025.070143)
Supplement: Supplementary file 5 [file OncolRes-34-70143-s005.docx]

Supplementary Figures

**
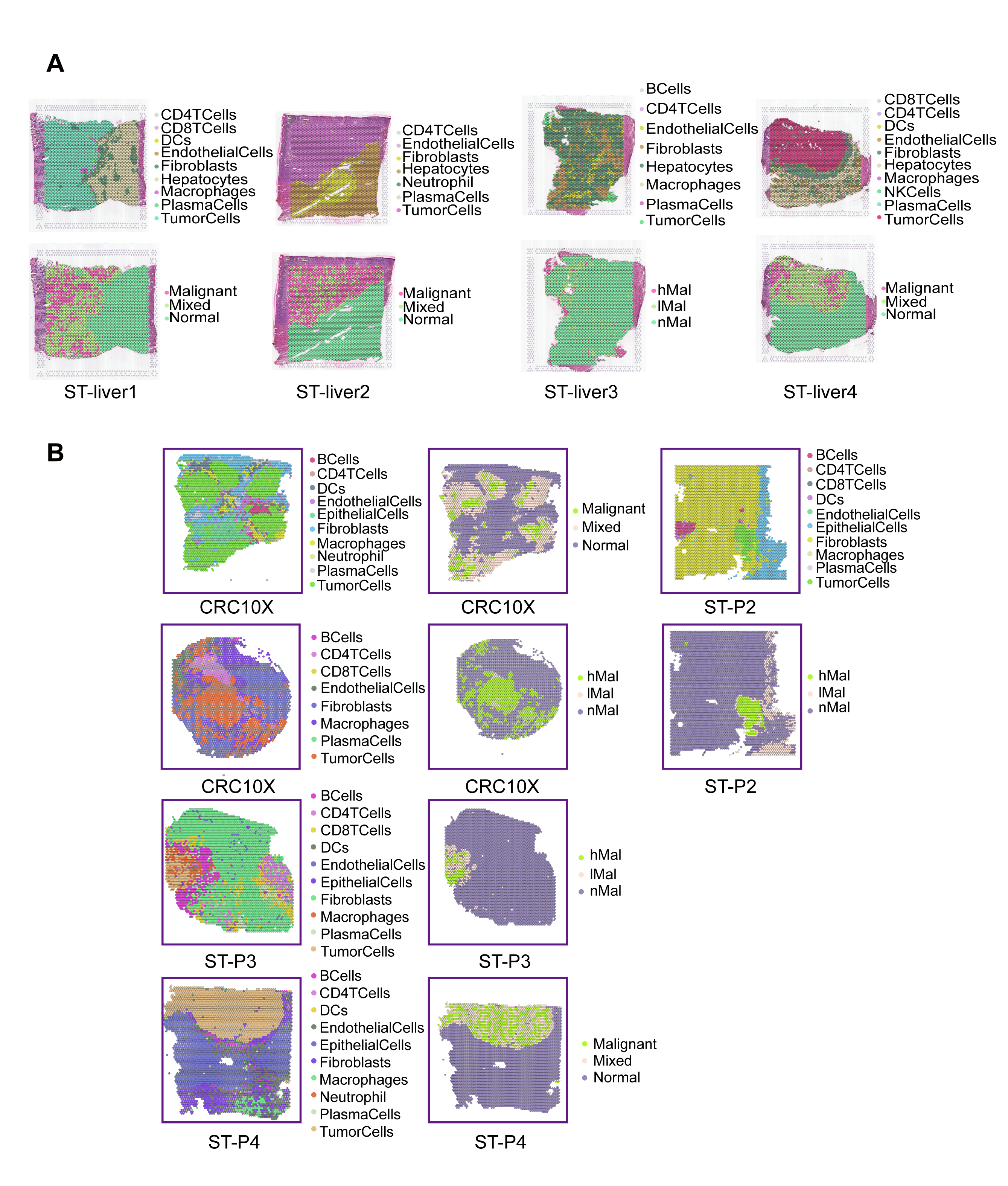
**

**Supplementary Figure S1.** Spatial transcriptomics reveals cell composition and TME heterogeneity in primary CRC and liver metastasis. (A) Spatial mapping of cell types and malignant regions in CRC liver metastasis (ST-liver1, ST-liver2, ST-liver3, ST-liver4). (B) Spatial analysis of cell types and malignancy states in primary CRC (CRC10X, ST-P2, ST-P3, ST-P4).

**
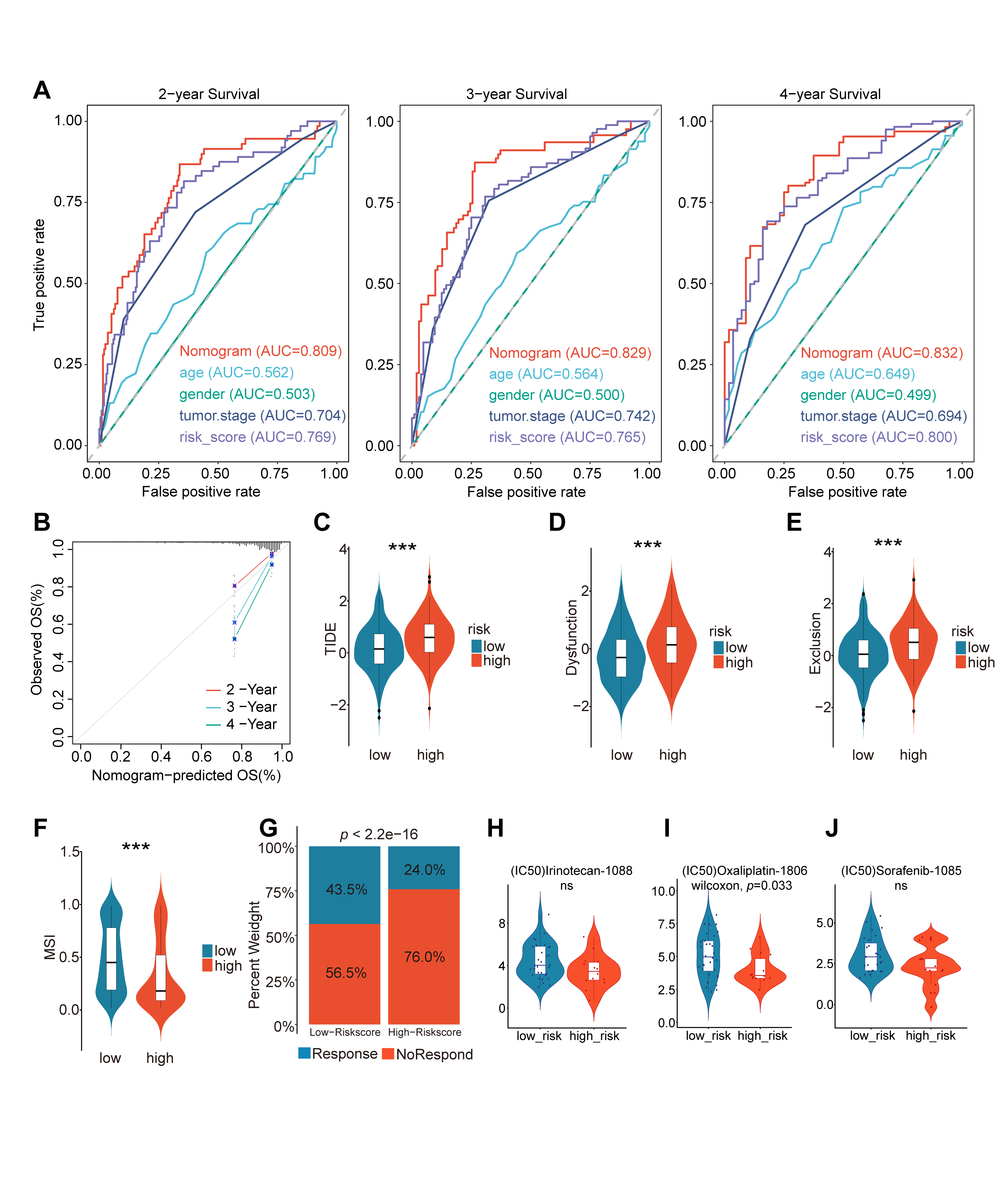
**

**Supplementary Figure S2.** Prognostic model assessment of survival prediction, TIDE, and drug sensitivity in CRC patients. (A) ROC curves of the constructed nomogram and other clinical features for predicting 2-year, 3-year, and 4-year OS. (B) Calibration curves of the constructed nomogram. (C–F) Differences in TIDE Characteristics between high- and low-risk groups. (G) Response rates to ICI treatment in high- and low-risk groups. (H–J) Comparison of IC_50_ values for common chemotherapy drugs between high- and low-risk groups. (ns, not significant; ****p* < 0.001)

**
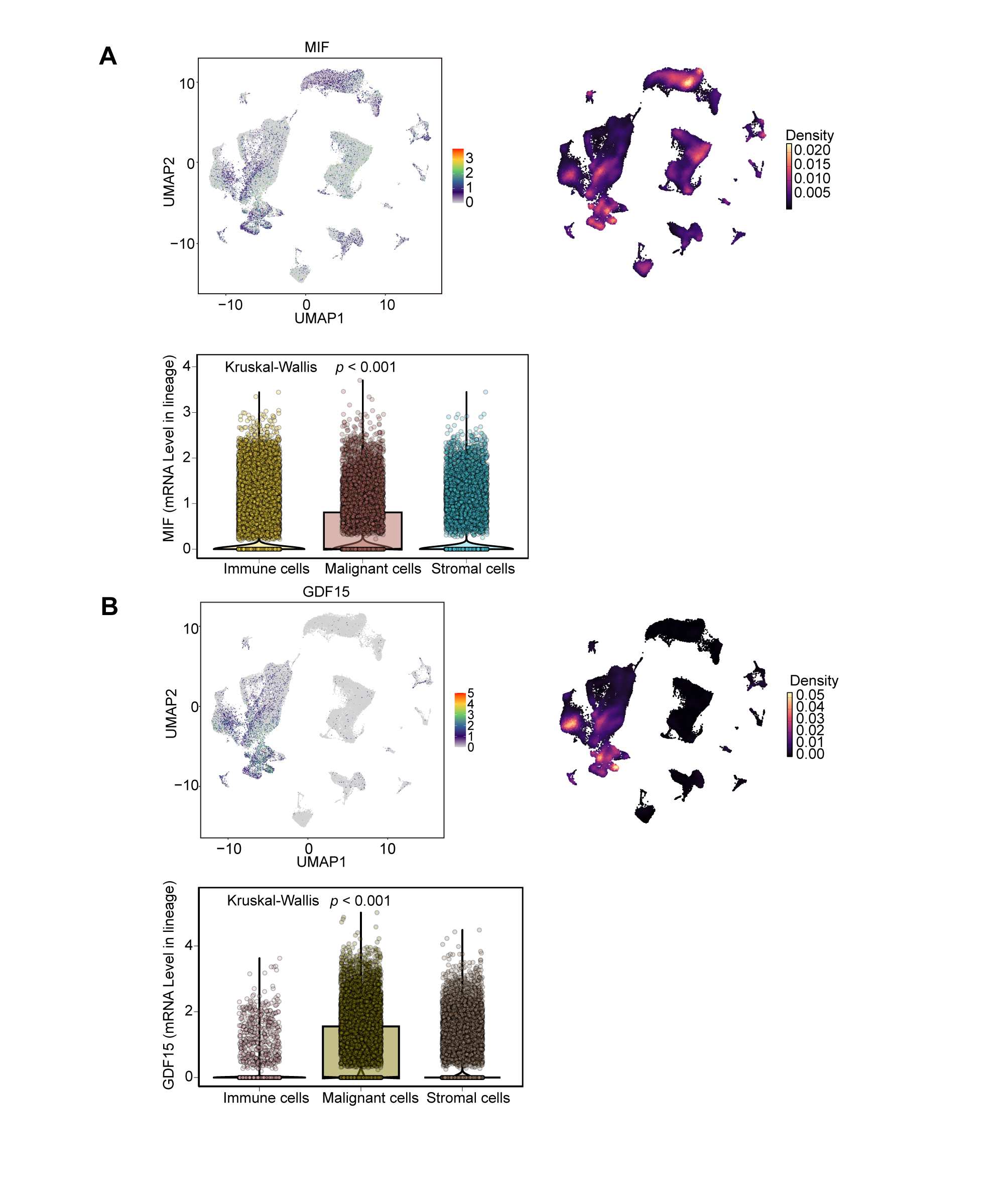
**

**Supplementary Figure S3.** Expression characteristics of MIF and GDF15 ligands in CRC single cells. (A) Single-cell expression analysis of macrophage migration inhibitory factor (MIF). (B) Single-cell expression analysis of growth differentiation factor 15 (GDF15).

**
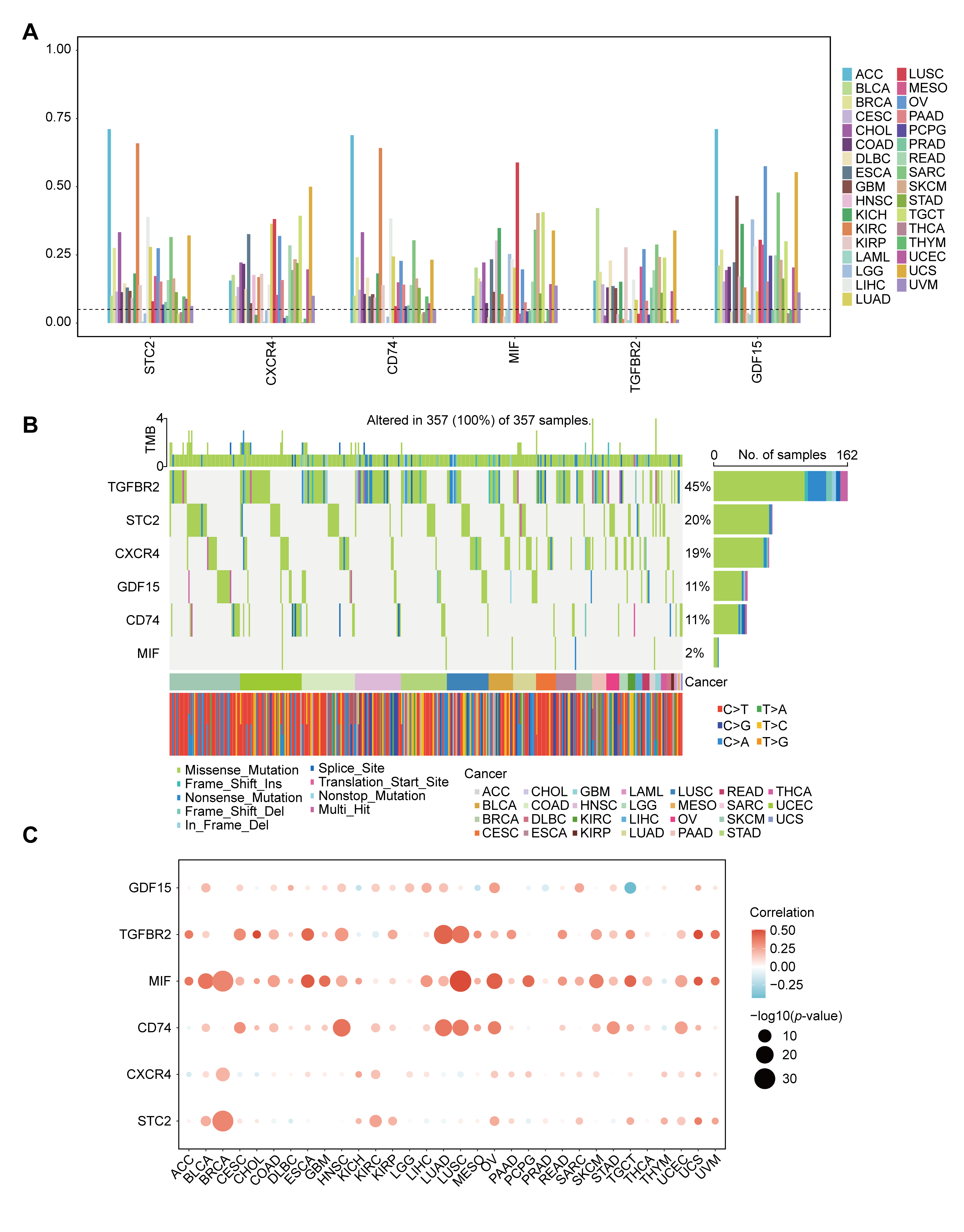
**

**Supplementary Figure S4.** Pan-cancer analysis reveals the landscape of genetic alterations and expression correlations of STC2, CXCR4, CD74, MIF, TGFBR2, and GDF15. (A) Gene copy number amplification ratio of the selected genes across different cancer types. (B) Oncoprint of somatic mutation distribution and SNV type classification for the selected genes. (C) Copy number and expression correlation of the selected genes.

## Supplementary Tables

**Table S3. Sequences of the qRT-PCR primers used in this study.**

| **Gene** | **Forward primer (5'-3')** | **Reverse primer (5'-3')** |
| --- | --- | --- |
| STC2 | TGAAATGTAAGGCCCACGCT | TTGAGGTAGCATTCCCGCTG |
| GAPDH | GATTTGGTCGTATTGGGCGC | TTCCCGTTCTCAGCCTTGAC |
